# Innovative approach to managing acromion fracture and acromioclavicular joint dislocation: A case report

**DOI:** 10.1016/j.ijscr.2024.109446

**Published:** 2024-03-05

**Authors:** Arash Maleki, Mohsen Dibamehr, Amir Bisadi, Emad Kouhestani

**Affiliations:** a Department of Orthopedic Surgery, Shahid Beheshti University of Medical Sciences, Tehran, Iran; b Bone Joint and Related Tissues Research Center, Akhtar Orthopedic Hospital, Shahid Beheshti University of Medical Sciences, Tehran, Iran

**Keywords:** Acromion fracture, Case report, Acromioclavicular joint, Dislocation, Scapular fracture

## Abstract

**Introduction:**

This study delves into the management of acromion fractures and acromioclavicular (AC) joint dislocations—orthopedic injuries with significant implications for shoulder function. Despite their infrequency, these injuries present challenges due to potential persistent pain and functional limitations. Current treatment strategies span from conservative measures to surgical interventions, yet there exists a notable gap in comprehensive data on specific surgical approaches.

**Presentation of case:**

We present a compelling case involving a 38-year-old male athlete who sought medical attention following a motor vehicle accident due to severe right shoulder pain. Upon admission to the emergency ward, the patient reported an inability to move the affected shoulder. Radiographic evaluations, comprising X-ray and computerized tomography scans, revealed a displaced fracture at the base of the acromion coupled with an AC dislocation. A novel surgical technique was employed, featuring coracoid fixation with mersilene thread and a 2-hole reconstruction plate—a distinctive approach in the field.

**Discussion:**

The systematic rehabilitation plan yielded successful healing and the restoration of normal shoulder function, offering promising insights into potential advancements in orthopedic practices.

**Conclusion:**

This case contributes valuable knowledge to the understanding of these complex injuries, paving the way for further exploration and refinement in their management. The innovative surgical approach showcased underscores the importance of continued research and exploration to enhance the overall treatment landscape for acromion fractures and AC joint dislocations.

## Highlights

• Novel surgical method for simultaneous management of acromion fractures and AC joint dislocations.
• Utilizes a widely available plate for acromion fixation in the surgical intervention.
• Pragmatic approach, considering financial considerations in healthcare and ensuring applicability in diverse settings.

## 1 Introduction

Acromion fractures and acromioclavicular (AC) joint dislocations are orthopedic injuries with substantial implications for shoulder function and overall musculoskeletal health [1]. The acromion, a prominent bony process of the scapula, plays a crucial role in maintaining shoulder stability and facilitating movement [1]. Fractures of the acromion and dislocations of the AC joint can result from various traumatic incidents, overuse, or underlying degenerative conditions [2]. These injuries are relatively rare but demand careful consideration due to their potential to cause persistent pain, restricted range of motion, and functional impairment [3]. Acromion fractures are uncommon, constituting roughly 8–16 % of scapular fractures, which, in turn, account for only 1 % of all fractures [3].

The treatment strategies for acromion fractures and AC joint dislocations are multifaceted, tailored to the specific characteristics of each injury. Acromion fractures may be managed conservatively through rest, immobilization, and physical therapy for milder cases, while more complex fractures often necessitate surgical intervention [4]. On the other hand, the treatment of AC joint dislocations varies based on the severity of the injury. Conservative measures, including rest, ice, and physical therapy, are typically employed for milder dislocations [4]. However, more severe cases may require surgical procedures [4]. Despite various reasons supporting the need for operative management in these fractures, there is a notable dearth of comprehensive data regarding the specific surgical approaches and fixation techniques employed in such cases.

In this study, we present a case with acromion fractures and AC joint dislocation to introduce a novel technique that promises to revolutionize the management of these injuries, offering a new perspective and potential advancements in the field of orthopedics.

## 2 Case presentation

We present the case of a 38-year-old male athlete who experienced right shoulder pain following a high energy trauma (motor vehicle accident (MVA)), involving an adducted arm with axial load to superior AC joint. Upon admission to the emergency ward, he reported severe pain and an inability to move the affected shoulder. The patient has no significant past surgical or medical history. Clinical examination revealed normal vital signs, and evaluation of the head, neck, chest, abdomen, and spine showed no abnormalities. Local physical examination and neurological assessment revealed normal findings. However, there was observable skin abrasion on the right proximal humerus. Tenderness was noted in the right AC joint, and further examination indicated limited range of motion in the right shoulder. The patient was unable to internally and externally rotate and elevate the right shoulder due to pain.

**Fig. 1:**
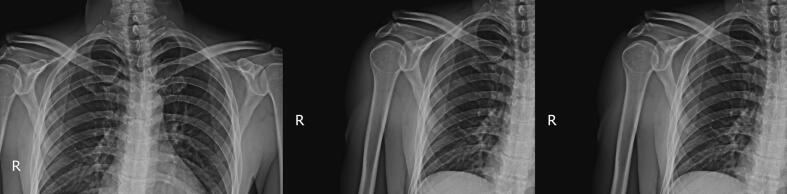
Preoperative X-ray indicating acromion fracture and AC dislocation.

**Fig. 2:**
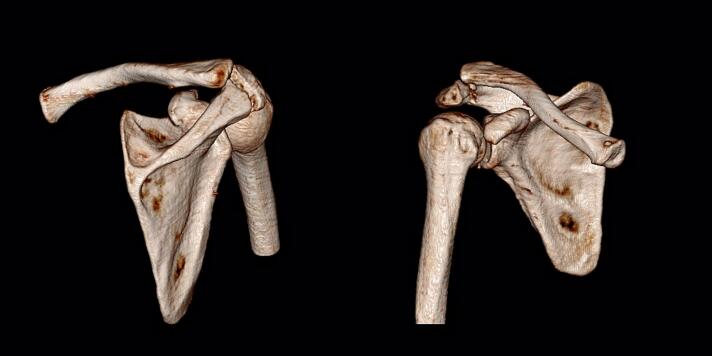
Preoperative CT scan indicating acromion fracture and AC dislocation.

Radiographic evaluations (X-ray and computerized tomography (CT) scan) revealed a displaced fracture at the base of the acromion and AC dislocation (Fig. 1, Fig. 2). The patient was scheduled for surgery the day after.

**Fig. 3:**
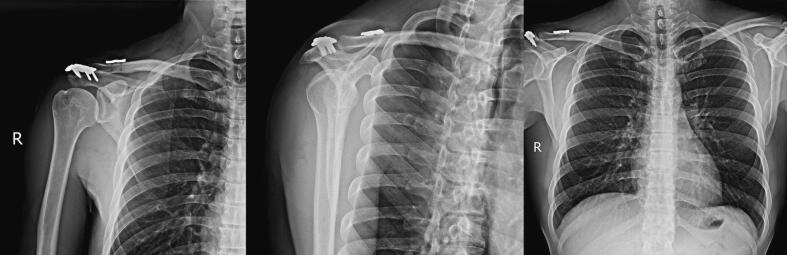
Postoperative X-ray.

The surgery was performed under general anesthesia, with the patient in a semi-sitting position. A direct longitudinal incision with a superior posterior distal approach of the clavicle, extending to the lateral side, was made. After exploring the AC joint and the fracture site of the acromion, the fracture was refreshed. The acromion base fracture was fixed with a 4-hole reconstruction plate (Fig. 3). The clavicle was stabilized to the coracoid using the Tight-Rope technique with mersilene thread and 2-hole reconstruction plate in order to prevent bone failure. Mersilene wire was utilized and fixed by wrapping it around the tip of the coracoid. The Tight-Rope technique involves twisting the Mersilene thread around the clavicle and passing it through tunnels inside the clavicle, then tying it to a 2-hole reconstruction plate. Instead of utilizing an endobutton, we used a 2-hole reconstruction plate. The stability of the superior and inferior aspects was checked, and it was deemed stable. Subsequently, the AC ligament was repaired with vicryl thread, and the anterior and posterior stability of the clavicle was verified. The platysma muscle was sewn, ensuring appropriate closure. Finally, the patient was transferred to the recovery room for post-operative monitoring and care.

A systematic rehabilitation plan tailored to address injuries of this type was implemented. The protocol included initiating active shoulder motion exercises after a two-week period, and six weeks later, the patient was able to resume nearly full activity without experiencing pain or discomfort. Radiographs conducted three months postoperatively indicated successful healing of the acromial fracture, with the AC joint maintaining congruency and a well-reduced state. By the six-weeks follow-up assessment, the patient had successfully returned to work, demonstrating normal shoulder function without any reported pain. Written informed consent for publication was obtained from the patient. This case report was has been reported in line with the Surgical CAse REport (SCARE) criteria [5].

## 3 Discussion

This paper introduces an innovative approach for addressing acromion fractures and AC joint dislocations. Our method involves repairing the AC joint dislocation, specifically the coracoid, through the implementation of the coracoclavicular (CC) ligament repair technique using mersilene thread and a 2-hole reconstruction plate.

The surgical intervention employed in our case, utilizing a direct longitudinal incision with a superior posterior distal approach of the clavicle, proved effective in addressing both the AC joint dislocation and the acromial base fracture. The meticulous steps, including refreshing the fracture site, coracoid fixation to the clavicle with a Tight-Rope technique, and stabilization with a 2-hole reconstruction plate, demonstrated a well-thought-out surgical strategy. The purpose of positioning the plate was to mitigate pressure elevation, thereby averting the risk of fractures. An alternative treatment option considered was the utilization of a clavicle hook plate; however, its implementation was avoided due to potential complications including shoulder pain, subacromial osteolysis, and subacromial fracture [6]. Instead, we selected the Tight-Rope technique without using endobutton, supported by evidence indicating superior functional recovery and diminished shoulder pain compared to the clavicle hook plate method [6].

Acromion fractures are relatively uncommon, typically occurring as part of scapular fractures, representing only 1 % of all reported fractures [4]. These fractures are primarily associated with high-speed traumas, such as MVA [7]. In contrast, AC joint separation is more prevalent than acromion fractures [8]. AC dislocation commonly arises from direct impacts or falls onto the shoulder [8]. In this process, the AC ligaments are initially torn, followed by the rupture of deltoid and trapezius muscle attachments [9]. The injury is finalized with the disruption of the CC ligaments, leading to AC joint dislocation [9]. It is noteworthy that concomitant acromion fractures are exceedingly rare in the context of this injury. This type of injury has been associated with a high incidence of peripheral neurological deficit such as complete axillary nerve palsy and with a poorer function [10]. In our case, the injury was the outcome of a MVA, which not only affected the AC joint but also resulted in a complete rupture of the AC capsule and CC ligaments. This, in turn, led to the fracture of the acromial base. However, it is noteworthy that the patient recovered without any complications and achieved a full range of motion, highlighting the effectiveness of our comprehensive treatment approach.

Acromion fractures are commonly classified based on the level of comminution and displacement [11]. Management strategies vary based on the type of acromion fracture [11]. One approach considers fracture displacement and accompanying ipsilateral shoulder injuries [11]. Surgical intervention is recommended for type 3 fractures, characterized by a reduction in subacromial space, as well as for symptomatic stress fractures and painful nonunions [12]. Studies propose surgical treatment for specific conditions related to acromion fractures, including symptomatic nonunion, subacromial impingement, displacement exceeding 1 cm, open fractures, and disruption of the superior shoulder suspensory complex (SSSC) [12]. These classifications and management recommendations provide a comprehensive framework for approaching acromion fractures, considering the nuances of displacement, comminution, and associated shoulder injuries.

The concomitant occurrence of an acromion fracture and AC joint dislocation presents a unique and intricate challenge in the field of orthopedic management. Historically, the treatment strategies for such complex shoulder injuries have lacked standardization, resulting in a variety of surgical techniques and rehabilitation protocols. Herein, we introduce a novel treatment method that seeks to streamline the management of these dual pathologies. Our innovative strategy involves a meticulous surgical intervention that not only addresses the acromion fracture but also focuses on the precise reconstruction of the AC joint. Unlike traditional approaches that treat these injuries distinctly, our method offers a comprehensive and unified solution. The incorporation of an accessible plate for acromion fixation, combined with a unique CC ligament repair technique using mersilene thread and a 2-hole reconstruction plate, distinguishes our approach from conventional methods. One of the distinctive features of our treatment strategy lies in its simultaneous addressing of both pathologies within a single surgical intervention. This minimizes the need for multiple procedures and contributes to a more efficient and cohesive approach to patient care.

Furthermore, our method recognizes the financial considerations in healthcare by incorporating the use of an accessible plate for acromion fixation. This pragmatic approach allows for effective treatment without compromising the quality of care. The unified rehabilitation protocol post-surgery is tailored to promote optimal recovery for both the acromion fracture and AC joint dislocation. This approach facilitates early mobilization and functional restoration, emphasizing long-term functional outcomes.

## Consent

The patient provided written informed consent for the publication of this case report and accompanying figures. Upon request, a copy of the written consent is accessible for examination by the Editor-in-Chief of the journal.

## Ethical approval

This study was reviewed and approved by the research committee of Shahid Beheshti University of Medical Sciences, Tehran, Iran on 1 December 2023. This study was also conducted in accordance with the Declaration of Helsinki.

## Funding

This research did not receive any specific grant from funding agencies in the public, commercial, or not-for-profit sectors.

## Guarantor

Arash Maleki, Emad Kouhestani.

## Research registration number

1. Name of the registry: NA
2. Unique identifying number or registration ID: NA
3. Hyperlink to your specific registration (must be publicly accessible and will be checked): NA.

## CRediT authorship contribution statement

**Arash Maleki:** Conceptualization, Investigation, Methodology, Project administration, Resources, Validation, Writing – original draft, Writing – review & editing. **Mohsen Dibamehr:** Conceptualization, Writing – original draft, Writing – review & editing. **Amir Bisadi:** Writing – review & editing. **Emad Kouhestani:** Conceptualization, Investigation, Project administration, Writing – original draft, Writing – review & editing.

## Declaration of competing interest

None.

## References

[1] Kuhn J.E., Blasier R.B., Carpenter J.E. (1994). Fractures of the acromion process: a proposed classification system. J. Orthop. Trauma.

[2] Babhulkar A., Pawaskar A. (2014). Acromioclavicular joint dislocations. Curr. Rev. Musculoskelet. Med..

[3] Joyce C.D., Seidl A.J. (2018). Managing acromial fractures: prevention and treatment, both nonoperative and operative. Annals of Joint..

[4] Frank R.M., Cotter E.J., Leroux T.S., Romeo A.A. (2019). Acromioclavicular joint injuries: evidence-based treatment. JAAOS-Journal of the American Academy of Orthopaedic Surgeons..

[5] Sohrabi C., Mathew G., Maria N., Kerwan A., Franchi T., Agha R.A. (2023). The SCARE 2023 guideline: updating consensus Surgical CAse REport (SCARE) guidelines. Int J Surg Lond Engl..

[6] Qi W., Xu Y., Yan Z., Zhan J., Lin J., Pan X., Xue X. (2021). The tight-rope technique versus clavicular hook plate for treatment of acute acromioclavicular joint dislocation: a systematic review and meta-analysis. J. Investig. Surg..

[7] Li X., Ma R., Bedi A., Dines D.M., Altchek D.W., Dines J.S. (2014). Management of acromioclavicular joint injuries. JBJS.

[8] Bahk M.S., Kuhn J.E., Galatz L.M., Connor P.M., Williams G.R. (2009). Acromioclavicular and sternoclavicular injuries and clavicular, glenoid, and scapular fractures. JBJS.

[9] Kurdy N.M., Shah S.V. (1995). Fracture of the acromion associated with acromioclavicular dislocation. Injury.

[10] Marsh J.L., Slongo T.F., Agel J., Broderick J.S., Creevey W., DeCoster T.A., Prokuski L., Sirkin M.S., Ziran B., Henley B., Audigé L. (2007). Fracture and dislocation classification compendium-2007: Orthopaedic Trauma Association classification, database and outcomes committee. J. Orthop. Trauma.

[11] Ogawa K., Naniwa T. (1997). Fractures of the acromion and the lateral scapular spine. J. Shoulder Elb. Surg..

[12] Hill B.W., Anavian J., Jacobson A.R., Cole P.A. (2014). Surgical management of isolated acromion fractures: technical tricks and clinical experience. J. Orthop. Trauma.

